# Effects of inquiry-based learning on hierarchical cognitive outcomes in tea cultural heritage education: a quasi-experimental study

**DOI:** 10.3389/fpsyg.2026.1849678

**Published:** 2026-09-18

**Authors:** Ying Long, Wee Hoe Tan, Rui Weng, Xuefen Li

**Affiliations:** 1 School of Design & Innovation, Fujian Jiangxia University, Fuzhou, China; 2 Faculty of Social Sciences & Liberal Arts, UCSI University, Kuala Lumpur, Malaysia; 3 College of Tea and Food Science, Wuyi University, Wuyishan, China; 4 College of Overseas Education, Minnan Normal University, Zhangzhou, China

**Keywords:** cognitive outcomes, heritage education, inquiry-based learning, tea cultural heritage, Bloom’s taxonomy

## Abstract

Understanding how instructional design shapes differentiated cognitive outcomes remains a central question in educational psychology, particularly in culturally grounded learning contexts where heritage knowledge must be retained, interpreted, and applied. Existing research on inquiry-based learning (IBL) in heritage education has relied largely on qualitative or non-comparative designs, and heritage cognition has typically been assessed through aggregate measures that may obscure important variation across cognitive levels. This study addresses these gaps by examining whether IBL produces differentiated cognitive effects across hierarchical levels in tea cultural heritage (TCH) education. A quasi-experimental design was employed with 66 seventh-grade pupils in the Wuyi Mountain area, Fujian Province, China, a UNESCO World Heritage site with deep historical ties to tea production. Two intact classes were assigned to different conditions: the experimental class received a purpose-designed IBL module structured around six inquiry stages—questioning, data collection, data analysis, result organization, sharing, and reflection—whereas the control class received conventional teacher-led instruction. Cognitive outcomes were assessed using a seven-level hierarchical framework adapted from Bloom’s taxonomy, spanning knowledge, comprehension, passive application, active application, analysis, synthesis, and evaluation. This framework was operationalized through a 100-point cognitive test combining multiple-response items for knowledge, comprehension, analysis, and evaluation with rubric-scored open-ended tasks for passive application, active application, and synthesis. Between-group and within-group differences were examined using non-parametric analyses. Results revealed a statistically significant overall cognitive advantage for the IBL condition, with a large effect size (*r* = 0.769). These gains were not uniformly distributed across the cognitive hierarchy. The strongest between-group contrasts emerged at application and synthesis (*r* = 0.816–0.831), where both groups had near-zero baseline scores. These immediate findings should be interpreted cautiously because the conditions differed in facilitation, context, and response format, and because the study involved two intact classes. The study demonstrates the value of hierarchical assessment, and provides quasi-experimental evidence concerning a contextually grounded IBL package in heritage education.

## 1 Introduction

Tea cultural heritage (TCH) education has become an increasingly important task in culturally embedded school contexts (UNESCO, 2003), particularly in regions where local heritage, formal schooling, and inter-generational transmission intersect. Wuyi Mountain in Fujian Province, China, which is both a World Heritage site and a major historical center of black tea and oolong tea, exemplifies this intersection. As a region possessing a dense concentration of heritage assets spanning tangible remains, intangible practices, and living cultural systems that continue to shape local livelihoods and community identity (Chen, 2025; Wagemakers, 2025), Wuyi Mountain creates conditions in which school learning can be connected to place-based cultural understanding. In 2022, traditional tea processing techniques and associated social practices in China were inscribed on the Representative List of the Intangible Cultural Heritage of Humanity. Local education policy has required schools to offer TCH-related courses or activities (Nanping Education Bureau, 2016). Yet the existence of such a mandate does not in itself guarantee the depth of learning it envisions.

The challenge of heritage education is not merely whether pupils are exposed to cultural content, but whether such exposure leads to structured and progressively deeper cognition. Existing research suggests a persistent gap between the richness of available heritage resources and the depth of learners’ cognitive engagement, particularly where heritage is present in the curriculum but not translated into structured opportunities for interpretation, inquiry, and meaning making (Wagemakers, 2025). As a result, pupils may learn to recognize tea varieties or recount historical episodes without becoming equally prepared to interpret heritage values, relate TCH to wider questions of development and sustainability, or develop the differentiated forms of understanding that heritage learning requires (Fontal et al., 2025). Prevailing instruction has often remained teacher-centered and knowledge-transmissive (Hein, 1998), operating through a model in which heritage content is presented by the teacher and reproduced by pupils. While adequate for conveying basic information, this approach is structurally mismatched with the epistemic demands of heritage cognition. It limits student agency, because when classroom activity is organized around teacher explanation and expected recall, learners have little opportunity to ask questions, interpret evidence, or construct meaning (Loyens et al., 2023). The result is not an absence of exposure, but a limitation in the depth and structure of pupils’ cultural understanding.

Inquiry-based learning (IBL) is relevant to this challenge because it shifts pupils from passive reception to active meaning-making in contextually grounded learning environments (Pedaste et al., 2015). As a student-centered approach, IBL engages learners in questioning, evidence gathering, interpretation, and reflection, thereby fostering forms of cognition that conventional instruction often leaves underdeveloped (Hein, 1998). This orientation is especially pertinent to heritage education, where knowledge is culturally situated, practically embedded, and inseparable from questions of meaning and value (Smith, 2006). The shift from teacher-as-authority to student-as-inquirer is a pedagogical response to the epistemic character of heritage itself: heritage meanings are historically layered, socially mediated, and open to interpretation in ways that call for active engagement rather than passive reception. Existing studies of IBL in heritage contexts have reported positive effects on engagement (Tsiviltidou and Vavoula, 2023), but have more rarely examined whether such gains are distributed across different levels of cognitive complexity in designs that allow for systematic comparison with conventional instruction.

A key gap in existing research is that heritage cognition is typically treated as a general outcome through aggregate measures, while its hierarchical structure remains under-examined. Yet heritage cognition is unlikely to be a unitary construct: it may involve factual recall, interpretive understanding, and evaluative judgment, each responding differently to pedagogy (Bloom et al., 1956). If instructional approaches produce gains at different cognitive levels, aggregate measures alone may conceal the most educationally meaningful dimension of their effects. To address this gap, the present study employs a non-equivalent control group pre-test/post-test quasi-experimental design (Shadish et al., 2002) to examine whether an IBL module improves pupils’ cognition of TCH and whether the intervention produces differentiated effects across hierarchical cognitive levels. The study was conducted with 66 secondary school pupils in Wuyi Mountain area, comparing an IBL module with conventional instruction across seven cognitive levels derived from Bloom’s taxonomy and adapted to the assessment of TCH learning. Two research questions guided the investigation:

*RQ1*: Does IBL significantly improve pupils’ overall cognition of TCH compared with conventional instruction?

*RQ2*: Does IBL produce differentiated effects across hierarchical levels of TCH cognition?

By disaggregating cognitive outcomes across hierarchical levels, this study moves beyond asking whether IBL works toward examining how its effects are distributed across the cognitive hierarchy. In doing so, it contributes not only comparative evidence on the cognitive effects of inquiry-based heritage education, but also a more differentiated account of what kinds of heritage cognition such pedagogy most strongly supports.

## 2 Literature review and conceptual framework

### 2.1 IBL in heritage education

IBL is a student-centered pedagogical approach in which learners construct knowledge through structured cycles of questioning, investigation, interpretation, and reflection (Pedaste et al., 2015). The approach shifts the locus of cognitive activity from teacher to student, foregrounds process alongside product, and typically operates through collaborative structures in which learners negotiate shared understandings (de Jong et al., 2024; Vygotsky, 1978). Meta-analytic evidence has linked inquiry-oriented instruction to gains in conceptual understanding and deeper processing, particularly when inquiry is appropriately scaffolded (Arifin et al., 2025; Lazonder and Harmsen, 2016).

The relevance of IBL to heritage education rests on a structural alignment between the demands of heritage learning and the cognitive processes that inquiry activates. Heritage knowledge is not simply a body of decontextualized facts; it is culturally situated, value-laden, and practically embedded in the lived experiences of communities (Achille and Fiorillo, 2022; Smith, 2006). Understanding a heritage domain requires interpretive engagement with questions of significance, continuity, and change—demands that align closely with inquiry processes of questioning, evidence gathering, and reflective meaning-making. This alignment is particularly pronounced in intangible cultural heritage education, where the objects of learning cannot be fully apprehended through textbook description alone but require contextual engagement and interpretive effort (Wagemakers, 2025). Studies in heritage-adjacent contexts have accordingly suggested that inquiry-oriented approaches can support engagement with heritage sites and deepen appreciation of cultural practices (Tsiviltidou and Vavoula, 2023; Van Doorsselaere, 2025).

Despite these encouraging indications, the evidence base for IBL in heritage education remains methodologically limited in three respects. First, much of the literature relies on qualitative case studies or practitioner accounts that document student experiences without systematic comparison to alternative instructional conditions (Cruickshank et al., 2026; Tsiviltidou and Vavoula, 2023). Second, controlled or quasi-experimental designs incorporating pre-post measurement and comparison groups remain rare in heritage education research (Menkshi et al., 2021; Santilli et al., 2025). Third, existing evaluations often treat cognition as a broad or aggregate outcome, making it difficult to determine not only whether inquiry-based interventions support more than basic knowledge acquisition, but also how their effects may be distributed across levels of cognitive complexity. Recent work in heritage cognition has begun to underscore the importance of more differentiated conceptions of knowing and understanding heritage, but level-sensitive assessment remains limited in intervention research (Fontal et al., 2025). This gap provides the rationale for combining an inquiry-based intervention, a comparative design, and level-sensitive cognitive assessment in the present study.

### 2.2 Hierarchical cognition in heritage learning

Heritage cognition involves the mental processes through which individuals acquire, organize, and use knowledge about heritage assets, their values, and related practices (Fontal et al., 2025). In educational settings, however, it has often been operationalized narrowly as factual knowledge about dates, production techniques, or site characteristics. A growing body of scholarship suggests that heritage learning involves a broader spectrum of cognitive engagement. At the lower end of this spectrum, learners acquire declarative knowledge such as names, dates, locations, and procedures that form the informational basis of heritage understanding. Yet meaningful heritage cognition also requires interpretive understanding, namely the capacity to explain why a heritage practice matters, to recognize its connections to broader cultural and ecological systems, and to appreciate tensions between preservation and development. At more advanced levels, heritage cognition entails evaluative judgment, including assessing conservation strategies, weighing competing values, and proposing contextually appropriate responses to heritage challenges (Achille and Fiorillo, 2022; Kisler, 2026). An intervention that improves factual recall without strengthening interpretive or evaluative capacities has achieved a fundamentally different educational outcome from one that promotes development across a broader cognitive range. Recognizing this distinction is essential for evaluating whether instructional approaches genuinely deepen heritage understanding or merely increase information retention.

The taxonomy of educational objectives proposed by Bloom et al. (1956) provides a widely adopted framework for conceptualizing such differentiation, ordering cognitive operations from knowledge and comprehension through application and analysis to synthesis and evaluation. Although Anderson and Krathwohl’s (2001) revision reconceptualized the taxonomy as a two-dimensional matrix, the original hierarchy retains practical value for assessment design focused on levels of cognitive processing. Its significance for the present study lies in providing a principled basis for distinguishing qualitatively different kinds of learning that an intervention may produce, rather than treating cognition as a single undifferentiated outcome. This distinction is especially relevant in heritage education, where the movement from knowing what a heritage asset is to understanding why it matters and how it should be governed represents a major pedagogical threshold.

Applying this framework to TCH assessment requires domain-specific adaptation. Drawing on the taxonomy proposed by Bloom et al. (1956) and adapting it to the assessment of TCH learning, the present study operationalizes a seven-level cognitive framework consisting of knowledge, comprehension, passive application, active application, analysis, synthesis, and evaluation. The distinction between passive and active application is pedagogically meaningful in heritage education because some tasks require pupils to apply knowledge in familiar or prompted situations, whereas others require more active transfer, adaptation, or problem-oriented use of that knowledge. In this sense, the distinction does not depart from the broader logic of application in Bloom’s taxonomy, but refines it in a way that is analytically useful for culturally embedded learning tasks. This seven-level structure enables a disaggregated analysis of learner performance across levels of cognitive complexity, thereby providing the analytical resolution required to address the gap identified above.

### 2.3 Integrative framework and hypotheses

To generate testable predictions about how IBL may influence hierarchical TCH cognition, the present study adopts an integrative conceptual framework drawing on three complementary theoretical perspectives: constructivism, situated cognition, and cooperative learning. These three perspectives were selected because they correspond to three distinguishable dimensions of the learning process that IBL activates: individual knowledge construction, contextual grounding of that knowledge, and social deepening through collaborative inquiry. The integrative claim is that no single theory is sufficient to explain how IBL may reshape heritage cognition across a hierarchy of levels; rather, it is their combined operation that provides a coherent basis for expecting both overall cognitive improvement and differentiated gains across the hierarchy.

Constructivist learning theory holds that knowledge is actively constructed through engagement with materials, problems, and prior experience rather than passively received (de Jong et al., 2024; Piaget, 1970; Vygotsky, 1978). In IBL, this principle is enacted through the inquiry cycle: pupils formulate questions, gather information, interpret evidence, and revise their understanding through reflection. For heritage cognition, this mechanism suggests that the depth of understanding depends on the degree of active cognitive engagement the instructional context demands. Cultural significance is not delivered as a fixed interpretation but actively assembled by learners as they relate evidence, practice, and context, supporting movement from factual acquisition toward more interpretive forms of understanding. Constructivism therefore accounts for what happens inside the individual learner: a shift from reception to construction.

Situated cognition theory holds that knowledge is shaped by, and inseparable from, the settings in which it is acquired and used (Brown et al., 1989; Cobb and Bowers, 1999; Lave and Wenger, 1991). Learning that occurs in culturally meaningful environments differs qualitatively from learning through decontextualized exposition. For TCH cognition, contextual grounding means that the significance of tea processing techniques or heritage landscapes is understood through direct encounter rather than textual summary alone. Contextually grounded knowledge carries richer associative structures, stronger links to lived practice, and greater potential to support interpretive and applied understanding, particularly in culturally immersive heritage learning contexts (Wagemakers, 2025). Situated cognition therefore accounts for how knowledge constructed through inquiry becomes culturally meaningful.

Cooperative learning theory holds that knowledge is refined through social interaction, particularly through explanation, negotiation, and the coordination of diverse perspectives (Ceballos et al., 2026). When learners articulate, compare, and defend understandings in dialogue with peers, cognitive demand rises from simple acquisition toward more complex interpretation, integration, and judgment. This social dimension is especially important for heritage cognition because heritage meanings are themselves socially constituted: the significance of a tea ceremony, the value of a conservation strategy, or the merits of a development proposal are matters of shared interpretation and reasoned judgment, not merely individual discovery.

Taken together, these three mechanisms are best understood as mutually reinforcing rather than parallel. Active construction gives learners something to work with cognitively, contextual grounding gives that construction cultural meaning, and collaborative inquiry deepens and tests emerging understanding through social negotiation. As illustrated in Figure 1, the IBL module is expected to influence hierarchical TCH cognition through the combined operation of these three theoretically distinct but functionally interrelated mechanisms. This combined operation supports two theoretical expectations that correspond to the two research questions of the study. First, pupils who learn through the IBL module will demonstrate greater overall cognitive improvement than pupils receiving conventional instruction, because IBL simultaneously activates construction, contextual grounding, and collaborative interpretation, mechanisms that transmissive instruction engages far less fully. By contrast, conventional transmissive instruction is more likely to generate gains through content exposure and factual familiarization, thereby supporting learning mainly where recognition and straightforward understanding are sufficient. Second, cognitive gains will not be uniformly distributed across the hierarchy. If foundational knowledge can be acquired to some degree through content exposure alone, while more interpretive and integrative forms of cognition depend more heavily on active construction, contextual grounding, and social negotiation, then the distribution of gains is unlikely to be uniform. The framework does not predict which specific level will exhibit the largest effect, but it does predict that different levels depend differently on the mechanisms activated by IBL, and that the contrast between IBL and conventional instruction will therefore be distributed unevenly across the cognitive hierarchy.

**Figure 1:**
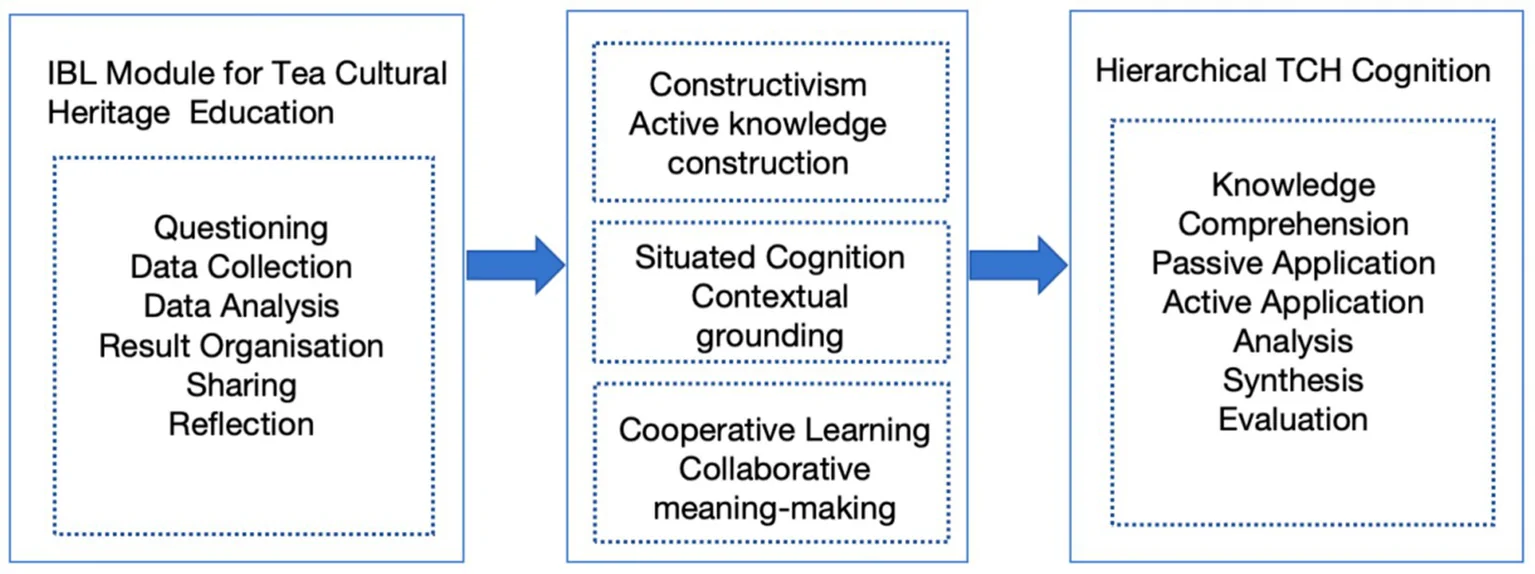
Integrative conceptual framework linking IBL and hierarchical TCH cognition.

These expectations generate two testable hypotheses:

*H1*: Pupils receiving the IBL module will demonstrate significantly greater improvement in overall TCH cognition than pupils receiving conventional instruction.

*H2*: The cognitive gains associated with the IBL module will vary across hierarchical levels of TCH cognition.

## 3 Methods

This study employed a non-equivalent control group pre-test/post-test quasi-experimental design. As shown in Figure 2, an experimental group receiving an IBL-based instructional package and a control group receiving conventional instruction completed identical cognitive assessments before and after the intervention period. Because allocation occurred at the level of two intact classes, the design, permits comparison of the two observed class conditions but does not isolate individual-level causal effects or permit a reliable estimate of cluster variance.

**Figure 2:**
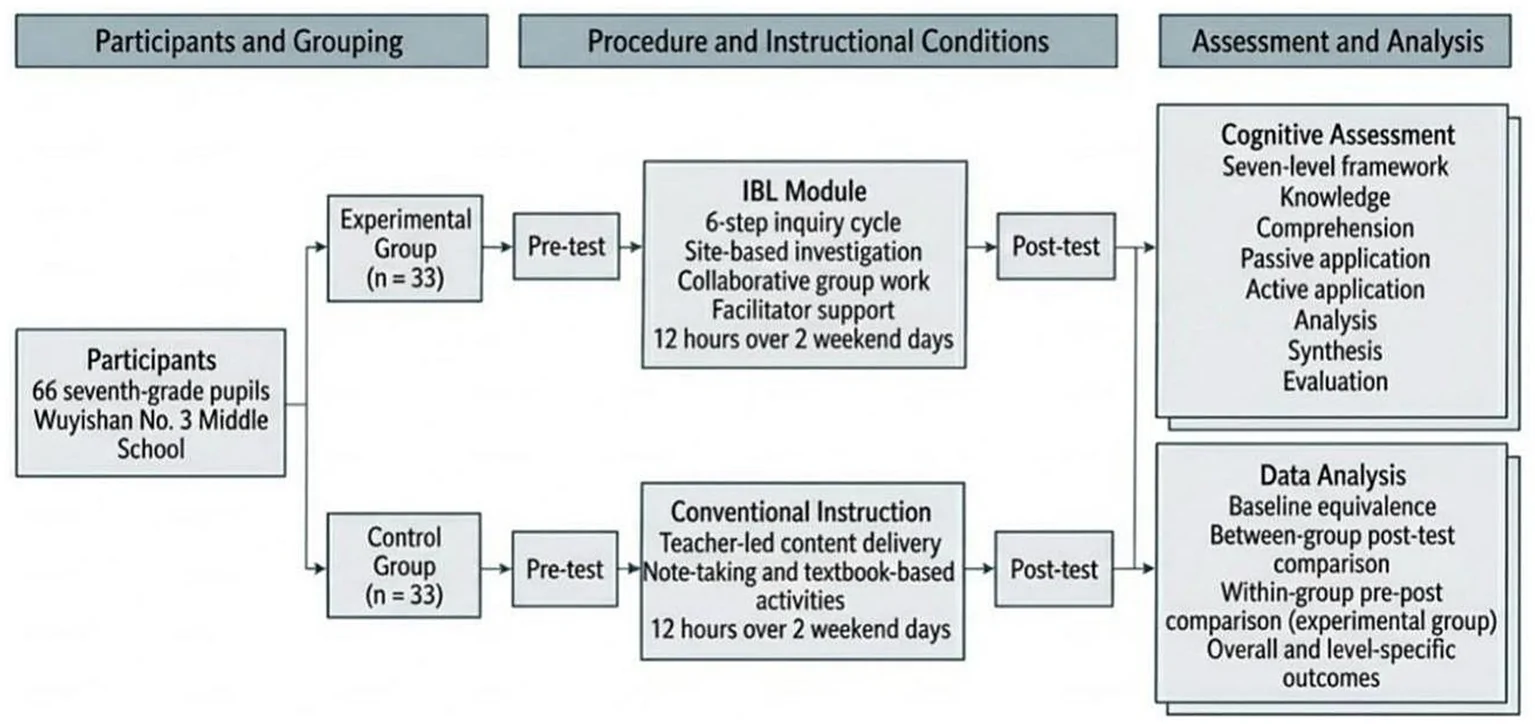
Overview of the research design and instructional intervention.

### 3.1 Participants

Participants were 66 seventh-grade pupils (aged 13–14) at Wuyishan No. 3 Middle School, Fujian Province, China. Two intact classes were assigned to the experimental (*n* = 33) and control (*n* = 33) conditions based on school administrative arrangement; random assignment at the individual level was not feasible. Demographic characteristics of the two groups are presented in Table 1, and baseline cognitive equivalence was verified through pre-test comparisons (see Results). Informed consent was obtained from all pupils and their parents or guardians.

**Table 1:** Demographic characteristics of participants.

Category	Characteristic	Experimental (*n* = 33)	Control (*n* = 33)
Age	13 years	22	14
	14 years	11	19
Gender	Male	17	16
	Female	16	17
TCH-related background	Born in Wuyi Mountain	25	23
	Born elsewhere	8	10
	Family member in TCH-related occupation	13	15
	Family member not in TCH-related occupation	20	18

### 3.2 Instructional intervention

The experimental group received instruction through a purpose-designed IBL module for TCH education, developed following the Sidek Module Development Model (Sidek and Jamaludin, 2005) and structured around a six-step inquiry cycle: questioning, data collection, data analysis, result organization, sharing, and reflection. Rather than treating these steps as separate classroom tasks, the module organized them as a continuous inquiry process. Pupils first generated questions about the cultural meaning, protection, and contemporary relevance of TCH. These questions then guided their evidence collection during visits to local heritage locations, including tea plantations, processing workshops, and historical tea trade routes. The materials gathered from these activities were subsequently discussed, compared, and interpreted within groups, enabling pupils to connect tea-related practices with broader cultural, historical, and ecological meanings. Each group then organized its findings into a coherent inquiry outcome, presented its understanding to peers, and reflected on how its interpretation of TCH had changed through the learning process. Module content was organized into four units: Unit 1 (fundamental knowledge and inquiry into the values of TCH), Unit 2 (critical thinking about TCH protection), Unit 3 (integrative activity on innovative application and dissemination of TCH), and Unit 4 (overall reflection and evaluation). The module underwent expert review by seven specialists and pilot testing with a comparable student group (n = 20) prior to implementation.

Pupils were organized into six inquiry groups of five to six, each supported by a trained facilitator. Facilitators were third-year undergraduate students from the Cultural Industry Management program at Fujian Jiangxia University, selected for their knowledge of cultural heritage management and familiarity with inquiry-based pedagogy. Before the intervention, they received structured training in the module procedures, group facilitation techniques, and their designated roles. Their function was to provide procedural support, including guiding pupils through the inquiry cycle, managing group activities, and maintaining the learning schedule, rather than delivering substantive content or directing pupils toward predetermined conclusions. To support consistency across groups, all facilitators followed the same module guide and procedural sequence throughout implementation. The module was implemented across two weekend learning days, totaling 12 h, with 6 h allocated to each day, in line with the school classification of heritage learning as an extracurricular activity.

The control group received conventional teacher-led instruction covering equivalent TCH content over a comparable intervention period. Instruction followed a transmissive format: the teacher presented heritage content through lectures, and pupils were expected to absorb and reproduce information through note-taking and textbook-based activities. The control condition covered the same broad thematic content as the IBL module, but did not involve structured inquiry cycles, collaborative group investigation, site-based exploration, or facilitated reflection. The experimental condition should be understood as an integrated IBL-based package; the present design cannot separate the contribution of inquiry pedagogy from that of small-group facilitation or field-based experience.

### 3.3 Instrument

Pupils’ cognition was assessed using a purpose-designed test aligned with the seven-level framework. The test comprised Section A (52 multiple-response multiple-choice items, 1 point each) and Section B (four open-ended questions, max 12 points each), for a total possible score of 100. Table 2 presents the alignment between cognitive levels and test structure.

**Table 2:** Cognitive test structure by hierarchical level.

Cognitive level	Section	Items	Format	Max score
Knowledge	A	A1–A26	Multiple-response MCQ (26 items × 1 pt)	26
Comprehension	A	A27–A33	Multiple-response MCQ (7 items × 1 pt)	7
Passive application	B	B1 (partial), B4 (partial)	Open-ended (rubric-scored)	12
Active application	B	B1 (partial), B4 (partial)	Open-ended (rubric-scored)	12
Analysis	A	A34–A44	Multiple-response MCQ (11 items × 1 pt)	11
Synthesis	B	B2, B3	Open-ended (rubric-scored)	24
Evaluation	A	A45–A52	Multiple-response MCQ (8 items × 1 pt)	8
Total				100

Multiple-response multiple-choice items assessed knowledge, comprehension, analysis, and evaluation. Open-ended items assessed passive application, active application, and synthesis. Questions B1 and B4 each comprised two scoring components: passive application (applying TCH knowledge in familiar contexts; max 6 points per question) and active application (creatively applying knowledge to novel situations; max 6 points per question). Questions B2 and B3 each assessed synthesis across three dimensions (max 4 points per dimension per question). The unequal maximum scores across levels reflect differences in response format rather than judgments about relative importance. Although the instrument included both selected-response and open-ended items, these were not intended to represent simple differences in difficulty. Rather, item format was aligned with the cognitive demands of each level. The selected-response items were designed as multiple-response questions rather than simple single-answer items, while open-ended items were used where application, transfer, or synthesis required generative responses. Expert review and pilot testing were used to check level alignment, clarity, age-appropriateness, and appropriate challenge across items before the main study.

Two trained raters scored all open-ended responses using detailed rubric criteria aligned with the hierarchical framework. Scores were reviewed jointly, and cases of disagreement were discussed with reference to the rubric until consensus was reached. The final analytic dataset retained the consensus scores only; independent pre-consensus ratings were not retained. Therefore, an ICC or Cohen’s kappa cannot be validly calculated retrospectively. Overall scores and level-specific scores were then calculated for each pupil. The same instrument was administered as both pre-test and post-test.

Content validity was established through review by a panel of seven experts with specialised knowledge in TCH, cultural heritage education, and inquiry-based pedagogy. The panel assessed alignment between test items and cognitive level specifications, clarity of language, and appropriateness for the target age group. Content validity index scores exceeded the recommended 70% threshold. A subject matter teacher moderated the test for curriculum alignment. The instrument was piloted with a comparable student group (*n* = 20); item difficulty and discrimination indices were calculated to refine items prior to the main study. The different item formats were selected to match the intended cognitive operations, but format remains aligned with cognitive level and may have contributed to the larger observed effects for open-ended application and synthesis tasks.

### 3.4 Procedure

Data collection proceeded in three phases: (1) both groups completed the pre-test under standardized conditions (100-min duration); (2) the experimental group completed the IBL module while the control group received conventional instruction, both scheduled across two weekend days; and (3) both groups completed the identical post-test under the same conditions immediately following the intervention period.

### 3.5 Data analysis

Baseline equivalence was assessed through Mann–Whitney *U* tests on pre-test scores. Between-group effects were then assessed using the Mann–Whitney *U* test on post-test scores, while within-group effects were assessed using the Wilcoxon signed-rank test on pre-to-post changes within the experimental group. The within-group analysis focused on the experimental group because the primary inferential interest concerned change associated with the IBL intervention; change in the control group is reported descriptively to contextualize the between-group contrast. Both analyses were conducted at the overall level and separately for each of the seven cognitive levels. Non-parametric methods were employed given the non-normal distribution of scores (Kolmogorov–Smirnov: K-S = 0.123, *df* = 66, *p* = 0.014) and the small sample size combined with the bounded score distributions observed in the data. Effect sizes were calculated as *r* = *z*/*√n* (Fritz et al., 2012) and interpreted using Cohen’s (1988) benchmarks (*r* = 0.10, small; *r* = 0.30, medium; *r* = 0.50, large). All analyses used *α* = 0.05. Because only two intact classes were available, these are unadjusted individual-score comparisons and should not be interpreted as cluster-adjusted estimates. The level-specific analyses were theoretically specified components of the hierarchical cognitive framework rather than *post hoc* exploratory tests. Nevertheless, the primary level-specific effects remained below a conservative Bonferroni-adjusted threshold (*α* = 0.007).

### 3.6 Research ethics statement

This study was conducted in accordance with applicable institutional ethical standards and the principles of the Declaration of Helsinki. The research protocol and study materials were reviewed and approved by the Academic Ethics Review Committee of Putian University in May 2023, prior to data collection in June 2023. The ethical review covered the study procedures, participant recruitment, data collection materials, and informed-consent arrangements. Because the participants were minors, written informed consent was obtained from their parents or legal guardians before participation. Participation was voluntary, and confidentiality and privacy were maintained throughout the research process.

## 4 Results

### 4.1 Baseline comparability

Prior to the intervention, Mann–Whitney *U* tests were conducted to compare pre-test cognitive scores between the experimental and control groups at the overall level and at each of the seven cognitive levels. Demographic characteristics of the two groups are presented in Tables 1, 3 reports the baseline cognitive comparisons.

**Table 3:** Baseline comparability: Pre-test cognitive scores by group.

Cognitive level	Max score	Experimental *M* (SD)	Control *M* (SD)	*p*
Knowledge	26	7.77 (2.97)	7.79 (2.80)	0.727
Comprehension	7	1.42 (1.15)	1.70 (1.24)	0.386
Passive application	12	0.30 (0.53)	0.33 (0.65)	0.906
Active application	12	0.06 (0.24)	0.06 (0.24)	0.906
Analysis	11	2.88 (1.47)	3.00 (1.39)	0.56
Synthesis	24	0.24 (0.56)	0.27 (0.67)	0.851
Evaluation	8	2.42 (1.64)	2.18 (1.31)	0.783
Overall cognition	100	15.30 (5.38)	15.33 (5.56)	0.964

All comparisons were conducted using the Mann–Whitney *U* test. No significant differences were found at any level (all *p* > 0.05).

No significant differences were found between the two groups at the overall level or at any of the seven cognitive levels (all *p* > 0.05), indicating comparable baseline cognitive profiles. Both groups also showed near-floor pre-test scores in passive application, active application, and synthesis, alongside generally low overall scores.

### 4.2 Effects of IBL on overall TCH cognition

Table 4 reports descriptive statistics for overall and level-specific scores across the two groups at pre-test and post-test. Figure 3 visualizes the corresponding score profiles as percentages of the maximum possible score for each level, while Table 5 presents the inferential results for between-group and within-group comparisons.

**Table 4:** Descriptive statistics for overall and hierarchical cognitive scores by group and time point.

Cognitive level	Max score	Control pre-test *M* (SD)	Control post-test *M* (SD)	Experimental pre-test *M* (SD)	Experimental post-test *M* (SD)
Overall cognition	100	15.33 (5.56)	25.91 (8.08)	15.30 (5.38)	50.79 (12.45)
Knowledge	26	7.79 (2.80)	11.64 (3.94)	7.77 (2.97)	16.06 (3.93)
Comprehension	7	1.70 (1.24)	2.64 (1.62)	1.42 (1.15)	4.33 (1.76)
Passive application	12	0.33 (0.65)	1.06 (0.83)	0.30 (0.53)	4.79 (1.80)
Active application	12	0.06 (0.24)	0.64 (0.74)	0.06 (0.24)	4.42 (1.70)
Analysis	11	3.00 (1.39)	4.91 (1.88)	2.88 (1.47)	6.94 (2.50)
Synthesis	24	0.27 (0.67)	1.70 (0.92)	0.24 (0.56)	9.33 (3.51)
Evaluation	8	2.18 (1.31)	3.39 (1.71)	2.42 (1.64)	4.91 (1.88)

*M*, mean; SD, standard deviation. Max score indicates the maximum possible score for each cognitive level.

**Figure 3:**
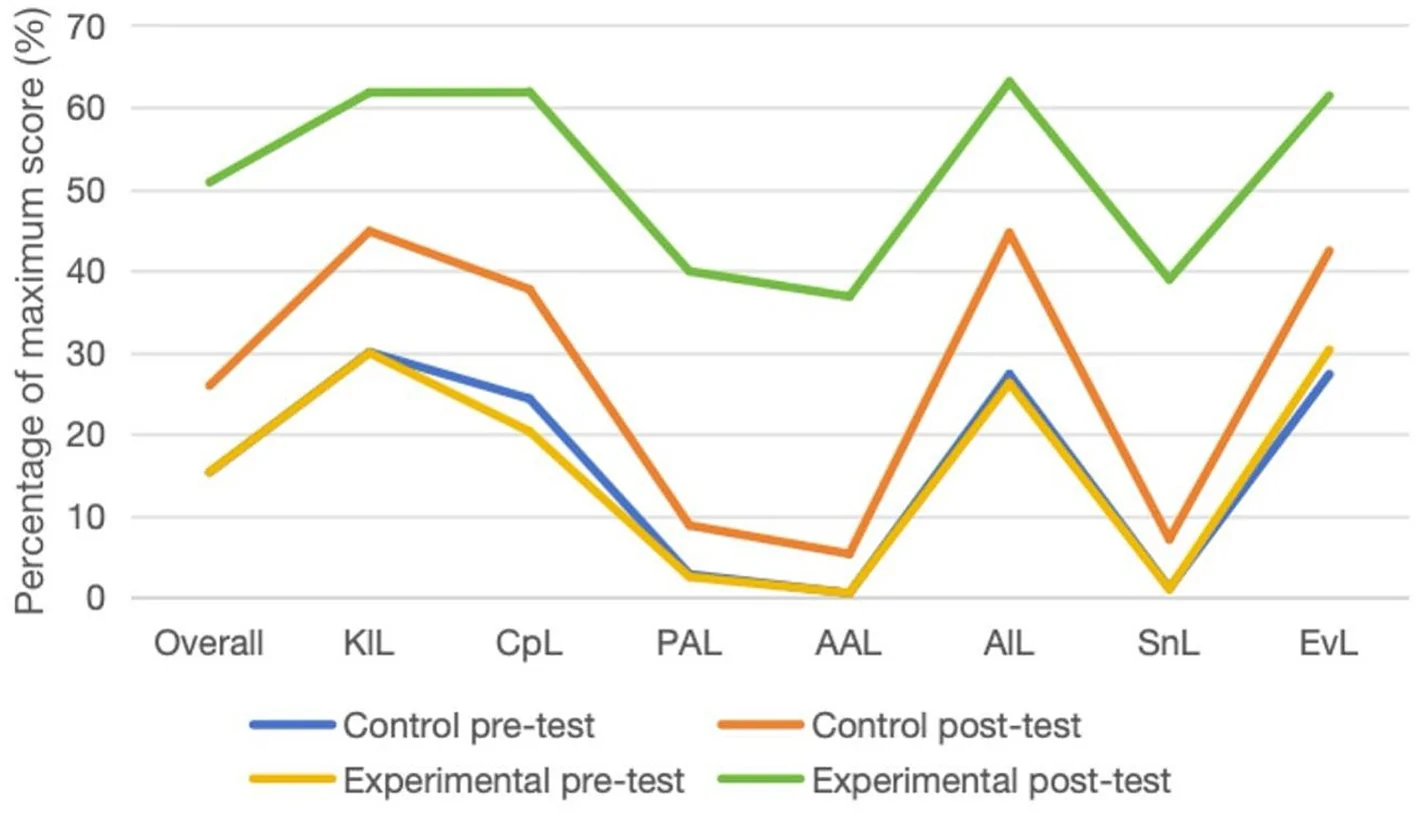
Mean score profiles across cognitive levels by group and time point. Scores are expressed as percentages of the maximum possible score for each cognitive level to allow comparison across outcomes with different score ranges. KIL, Knowledge, CpL, Comprehension, PAL, Passive Application, AAL, Active Application, AIL, Analysis, SnL, Synthesis, and EvL, Evaluation.

**Table 5:** Inferential statistics for between-group and within-group comparisons.

Cognitive level	Between-group (post-test)			Within-group (experimental group)		
	*z*	*p*	*r*	*z*	*p*	*r*
Overall cognition	−6.250	<0.001	0.769	−5.014	<0.001	0.872
Knowledge	−4.066	<0.001	0.500	−5.018	<0.001	0.874
Comprehension	−3.671	<0.001	0.452	−4.981	<0.001	0.867
Passive application	−6.631	<0.001	0.816	−5.032	<0.001	0.876
Active application	−6.647	<0.001	0.818	−4.964	<0.001	0.864
Analysis	−3.277	<0.001	0.403	−5.032	<0.001	0.876
Synthesis	−6.749	<0.001	0.831	−5.018	<0.001	0.873
Evaluation	−3.147	0.002	0.387	−4.821	<0.001	0.838

Between-group comparisons were conducted using the Mann–Whitney *U* test on post-test scores. Within-group comparisons were conducted using the Wilcoxon signed-rank test on pre- to post-test scores in the experimental group. Effect sizes are reported as *r* = *z*/*√n*.

As shown in Table 4 and Figure 3, both groups showed higher overall post-test scores, but the increase was substantially greater in the experimental group.

Table 5 confirms a statistically significant between-group post-test difference with a large effect size (*z* = −6.250, *p* < 0.001, *r* = 0.769), alongside a significant pre-to-post gain in the experimental group (*z* = −5.014, *p* < 0.001, *r* = 0.872). Together, these results support H1.

### 4.3 Effects of IBL across hierarchical cognitive levels

The effects of the IBL condition were not distributed evenly across the seven hierarchical cognitive levels. Table 4, Figure 3, Table 5, and Figure 4 show that all between-group post-test comparisons were significant, but the magnitude of the contrasts varied by level.

**Figure 4:**
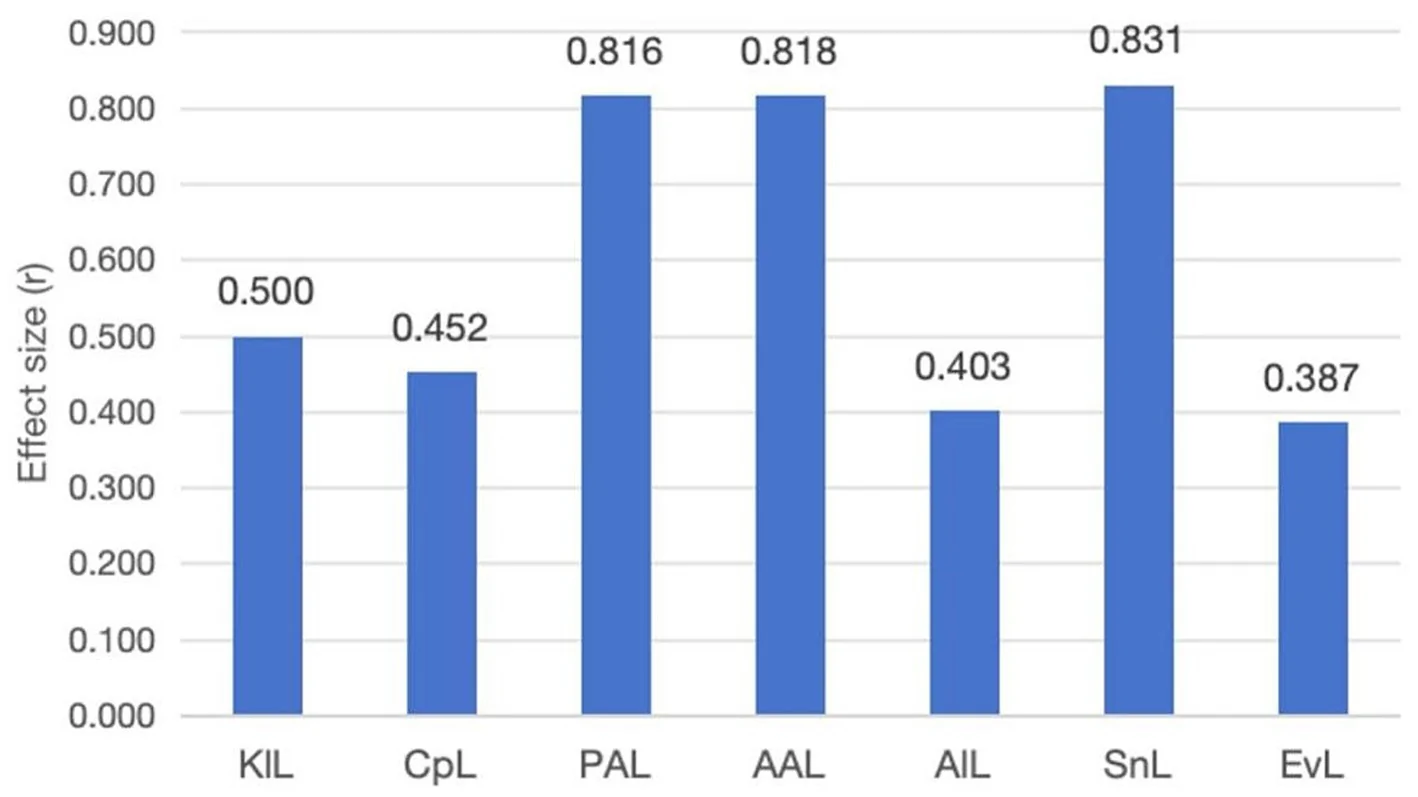
Between-group effect sizes across the seven hierarchical cognitive levels. Bars represent effect sizes (*r*) for post-test differences between the experimental and control groups. Reference lines indicate Cohen’s (1988) benchmarks for small (*r* = 0.10), medium (*r* = 0.30), and large (*r* = 0.50) effects. KIL, Knowledge, CpL, Comprehension, PAL, Passive Application, AAL, Active Application, AIL, Analysis, SnL, Synthesis, and EvL, Evaluation.

The strongest between-group effects were observed at passive application (*r* = 0.816), active application (*r* = 0.818), and synthesis (*r* = 0.831). As Figure 4 illustrates, these three levels were clearly distinguished from the others in effect size magnitude. At these levels, both groups entered the study with near-zero baseline scores, but the post-test performance of the experimental group diverged sharply from that of the control group. The experimental group reached 40.0, 36.8, and 38.9% of the maximum score in passive application, active application, and synthesis, respectively, whereas the control group remained at 8.8, 5.3, and 7.1%. These levels therefore represent the domains in which the contrast between IBL and conventional instruction was greatest.

Knowledge (*r* = 0.500) and comprehension (*r* = 0.452) showed substantial but less differentiated between-group effects. Both groups increased at these foundational levels, but the contrast between conditions was less pronounced than that observed at the application and synthesis levels.

Analysis (*r* = 0.403) and evaluation (*r* = 0.387) showed comparatively smaller between-group effects, though both remained statistically significant. At these levels, the experimental group still outperformed the control group clearly, but the contrast between conditions was weaker than that observed at the application and synthesis levels.

Because effect size r reflects distributional separation between groups rather than absolute score attainment, effect size magnitude does not necessarily correspond directly to post-test score percentages. Percentage values are therefore reported to contextualise the absolute level of performance at each tier, whereas *r* values indicate the strength of the between-group contrast.

Overall, the level-specific findings indicate that the cognitive gains associated with the IBL module were distributed unevenly across the seven hierarchical levels. The strongest between-group contrasts appeared at the application and synthesis levels, whereas knowledge, comprehension, analysis, and evaluation showed comparatively smaller—though still significant—advantages for the IBL condition. This pattern supports H2.

## 5 Discussion

### 5.1 Interpreting the overall cognitive advantage of the IBL condition

The IBL-based instructional package was associated with significantly higher immediate overall cognitive scores than conventional instruction. This pattern is compatible with deeper cognitive engagement with TCH, but it should not be attributed solely to inquiry pedagogy because facilitation and field-based experience were integral to the experimental condition.

This finding is broadly consistent with prior evidence that IBL supports stronger cognitive outcomes than transmissive instruction (Lazonder and Harmsen, 2016; Loyens et al., 2023). At the same time, heritage education research has often relied on qualitative accounts, small-scale case studies, or undifferentiated outcome measures, which has limited the strength of causal claims about inquiry-based effects in this field (Van Doorsselaere, 2025; Wagemakers, 2025). The present study helps address this gap by providing quasi-experimental evidence from a culturally embedded heritage context using a domain-specific hierarchical assessment, a combination that has remained insufficiently represented in prior research.

The overall advantage can be interpreted through the integrative framework developed in this study. The module simultaneously supported knowledge construction through the inquiry cycle, contextual grounding through site-based investigation, and collaborative deepening through group inquiry and reflective presentation. Rather than receiving TCH content as predetermined information, pupils in the experimental condition were required to formulate questions, gather and interpret evidence, and work with culturally embedded materials in a socially interactive learning process. This design is consistent with constructivist accounts of learning as active meaning-making rather than passive reception (Piaget, 1970; Vygotsky, 1978), with situated views of knowledge as shaped by the contexts in which it is encountered and used (Brown et al., 1989), and with broader evidence that IBL supports deeper processing and, under supportive conditions, higher-order thinking (Arifin et al., 2025). While transmissive instruction can deliver content effectively at the level of factual information, it does not systematically require pupils to formulate questions, gather evidence, construct interpretations, or negotiate understandings with peers. The convergence of construction, contextualization, and collaboration within the IBL approach therefore provides a plausible explanation for the substantial overall advantage observed.

An important qualification is that the control group also showed higher post-test mean scores than pre-test mean scores, indicating that conventional instruction was not cognitively irrelevant. Structured content exposure can support gains in factual familiarity and basic understanding, and the improvement observed in the control group is consistent with that expectation. However, the much larger gains under the IBL condition suggest that content exposure alone was insufficient to produce the level of cognitive development associated with IBL. The difference between the two conditions therefore lies not simply in whether score change occurred, but in the depth, scale, and structure of the cognitive performance differences observed under instructional condition.

### 5.2 Interpreting the differentiated pattern across cognitive levels

One of the most theoretically consequential findings is that the cognitive effects of the IBL module were not distributed uniformly across the hierarchy. The strongest between-group contrasts appeared at application and synthesis, intermediate effects at knowledge and comprehension, and comparatively smaller effects at analysis and evaluation. This pattern has important implications for how heritage cognition is conceptualized and how instructional effects are evaluated.

The differentiated pattern supports the argument that heritage cognition is not a single undifferentiated construct, but a structured hierarchy of qualitatively different operations. Bloom’s taxonomy serves here not simply as a classificatory device, but as a framework for revealing educationally meaningful differences in the kinds of learning that interventions produce (Anderson and Krathwohl, 2001; Bloom et al., 1956). Had cognition been assessed only as an aggregate score, one of the most important findings of the study would have remained hidden: IBL did not simply produce “more cognition”, but a distinctive profile of gains whose distribution itself carries theoretical significance. This finding lends support to treating the hierarchical structure of heritage cognition as analytically consequential, rather than as a purely classificatory device.

The especially strong effects at application and synthesis warrant cautious interpretation. At these levels, both groups entered the study with near-zero baselines, yet only the IBL condition produced substantial growth. These levels require learners to mobilize heritage knowledge rather than merely retain it—using it in practical contexts, transferring it to unfamiliar situations, and integrating multiple elements into coherent outputs. Data collection in authentic heritage settings creates conditions for application (Lazonder and Harmsen, 2016), while group-based analysis, presentation, and reflection require pupils to coordinate and integrate what they have learned—precisely the operations that define synthesis. However, these outcomes were assessed with open-ended tasks, whereas several other levels used multiple-response items. The alignment of item format with cognitive level, together with the near-floor baseline, may have amplified between condition separation; the results therefore indicate performance on this assessment rather than a format-independent estimate of cognitive capacity.

The particularly strong effect at the synthesis level may also reflect the communicative demand of the sharing phase. Pupils were not only asked to collect and organize information, but also to present inquiry outcomes in ways that made dispersed observations intelligible to others. This requirement likely pushed them beyond fragmentary findings toward more coherent integration of heritage meanings. In a heritage context, where meaning often emerges through the connection of practice, place, value, and history, such communicative integration may be especially consequential. This interpretation is consistent with the view that heritage learning becomes educationally consequential when learners move beyond exposure and engage in locally grounded inquiry through which cultural knowledge is actively interpreted rather than passively received (Van Doorsselaere, 2025).

Knowledge and comprehension showed a different pattern. Both conditions produced notable gains at these foundational levels, and the between-group differential was therefore narrower. This is consistent with the expectation that factual recall and basic understanding can be supported, to some degree, through structured content exposure alone (Arifin et al., 2025). The inquiry-based condition still outperformed conventional instruction at these levels, but the contrast was less dramatic where both pedagogies could plausibly foster improvement—a pattern that aligns with broader review evidence linking IBL to stronger conceptual understanding even at foundational cognitive levels (Lazonder and Harmsen, 2016).

Analysis and evaluation showed significant but comparatively smaller between-group effects. This should not be read as evidence that the IBL module was ineffective at these levels. Rather, the smaller contrasts likely reflect the fact that these levels involve more demanding operations, including decomposing relationships, weighing competing considerations, and making reasoned judgments. In the TCH context, they also require pupils to work through tensions between preservation and development, cultural value and practical change—tasks that place heavier cognitive demands on lower-secondary learners than either application or synthesis. The significant within-group gains observed in the experimental group nonetheless indicate that the IBL-based package was associated with improvements in these higher-level performance outcomes. Critical thinking of this kind is known to be shaped by implementation conditions, scaffolding, and the cognitive structure of learning tasks (Loyens et al., 2023). These capacities may require more sustained practice, more explicit metacognitive support, and more recursive engagement than a 12-h intervention could provide. This pattern is consistent with broader evidence that critical thinking and evaluative reasoning develop incrementally rather than through brief, isolated exposure (Abrami et al., 2015).

Taken together, the differentiated pattern suggests that the cognitive effects of inquiry-based pedagogy are selective rather than monotonic. The strongest comparative advantage appeared not at the top of the hierarchy in a simple “higher-order thinking” sense, but at the point where learners had to move from retention to mobilization and integration. In this respect, the study refines general claims about IBL by identifying the cognitive transition at which its contribution appears most distinctive in a heritage education context.

### 5.3 Implications for theory and pedagogy

The findings carry implications at both the theoretical and pedagogical levels. A first implication is that heritage cognition should be treated as empirically differentiated rather than unitary. The present study shows that intervention effects on factual recall may differ substantially from those on application, synthesis, or evaluation. Aggregate scores can mask important variation in cognitive profiles, and hierarchical assessment is therefore not merely a taxonomic refinement, but an important strategy for identifying the most educationally meaningful variation in instructional outcomes.

A second implication concerns the role of cultural embeddedness. Pupils in both groups lived in the Wuyi Mountain area and had everyday proximity to TCH, yet both entered the study with near-zero performance at several of the more demanding levels. What activated those capacities was not cultural proximity itself, but the pedagogical structuring of that proximity through inquiry. As Brown et al. (1989) argue, knowledge is shaped by the contexts in which it is used; cultural embeddedness becomes cognitively productive only when transformed into organized opportunities for investigation, interpretation, and collaborative meaning-making (Achille and Fiorillo, 2022; Wagemakers, 2025). For heritage education, this suggests that the issue is not curricular presence alone, but pedagogical realization.

A third implication is that the comparative advantage of IBL in heritage education may be most pronounced at the point of knowledge mobilization and integration. Rather than elevating all cognitive levels equally, the module appeared especially effective where pupils had to use, transfer, and synthesize cultural knowledge. This sharpens broader claims about IBL by suggesting that its strongest contribution lies not simply in promoting higher-order thinking in general, but in helping learners move from knowing heritage content to actively working with it.

At the same time, the comparatively smaller effects at analysis and evaluation suggest that IBL may need supplementary pedagogical support when advanced evaluative reasoning is the target. This is consistent with evidence that IBL can support higher-order thinking under appropriate conditions (Arifin et al., 2025). More specifically, recent review work suggests that such effects are shaped by how tasks are sequenced, how reasoning is scaffolded, and how learners are supported in making and defending judgments (Loyens et al., 2023). This does not diminish the value of IBL; rather, it suggests that its strengths may be complemented by more explicitly scaffolded instructional designs when the most demanding forms of judgment are at stake. From a practical standpoint, heritage content should be organized around inquiry tasks, learning activities should create opportunities for application and synthesis, and assessment should be aligned with differentiated cognitive outcomes. The weekend-only implementation in the present study also suggests that more sustained curricular embedding may strengthen the cognitive impact of heritage interventions. Where heritage education aims to cultivate contextually grounded and interpretively active engagement with cultural content, these findings suggest that pedagogy needs to be designed to support more than exposure alone.

## 6 Conclusion

This study examined whether an IBL module could improve secondary school pupils’ cognition of TCH and whether its cognitive effects varied across hierarchical levels. Using a quasi-experimental design with 66 pupils in the Wuyi Mountain area, the study compared an IBL intervention with conventional instruction across seven cognitive levels adapted from Bloom’s taxonomy.

Two principal findings emerged. First, the IBL-based instructional package was associated with a statistically significant overall advantage over conventional instruction. Second, the largest unadjusted between-group contrasts appeared at application and synthesis. Given the short duration, near-floor baselines, different assessment formats, and class-level allocation, these results should be interpreted as immediate, context-bound differences rather than evidence of large, isolated, or durable effects of IBL.

These findings contribute to the literature in three ways. Empirically, the study provides preliminary quasi-experimental evidence from cultural heritage education, a literature that has often relied on qualitative or non-comparative designs. Methodologically, it demonstrates the value of assessing heritage cognition hierarchically rather than relying on aggregate scores alone. Theoretically, it suggests that an IBL-based instructional package may be particularly relevant to tasks requiring application and synthesis, subject to the methodological qualifications noted above.

Several limitations qualify these conclusions. The quasi-experimental design involved two intact classes, so the individual-score analyses do not account for cluster effects and cannot establish causality. The experimental condition also combined inquiry pedagogy with a lower facilitator-to-student ratio and field-based tea-plantation and processing-workshop experiences; these contextual and attentional differences are threats to internal validity and prevent attribution of the observed differences to IBL alone. Application and synthesis were assessed through open-ended responses, whereas several other levels used multiple-response items. Thus, response format and the near-floor pre-test scores at these levels may have contributed to the large effect sizes. Although two trained raters used detailed rubrics and reached consensus, independent pre-consensus ratings were not retained in the final analytic dataset; an ICC or Cohen’s kappa cannot therefore be reconstructed retrospectively. The sample was drawn from a single school, no delayed post-test was administered, and the 12-h intervention measured immediate outcomes only. The effects should consequently be regarded as preliminary and context-bound.

Future research should therefore test these findings with larger and more diverse samples, stronger comparison designs where feasible, and longitudinal follow-up to examine the persistence of cognitive gains. More sustained interventions embedded in regular instructional schedules may be especially important for clarifying how inquiry-based pedagogy supports the development of analysis and evaluation over time. More broadly, the findings suggest that if cultural heritage is to remain educationally meaningful for younger generations, pedagogy needs to move beyond passive exposure toward structured inquiry and deeper understanding.

## Data Availability

The raw data supporting the conclusions of this article will be made available by the authors, without undue reservation.
